# Selective blood-nerve barrier leakiness with claudin-1 and vessel-associated macrophage loss in diabetic polyneuropathy

**DOI:** 10.1007/s00109-021-02091-1

**Published:** 2021-05-21

**Authors:** Adel Ben-Kraiem, Reine-Solange Sauer, Carla Norwig, Maria Popp, Anna-Lena Bettenhausen, Mariam Sobhy Atalla, Alexander Brack, Robert Blum, Kathrin Doppler, Heike Lydia Rittner

**Affiliations:** 1 Center for Interdisciplinary Pain Medicine, Department of Anesthesiology, University Hospital of Würzburg, 97080 Würzburg, Germany; 2 Institute of Clinical Neurobiology, University Hospital of Würzburg, 97078 Würzburg, Germany; 3 Department of Neurology, University Hospital of Würzburg, 97080 Würzburg, Germany

**Keywords:** Neuropathy, Barrier, Pain, Macrophages

## Abstract

**Abstract:**

Diabetic polyneuropathy (DPN) is the most common complication in diabetes and can be painful in up to 26% of all diabetic patients. Peripheral nerves are shielded by the blood-nerve barrier (BNB) consisting of the perineurium and endoneurial vessels. So far, there are conflicting results regarding the role and function of the BNB in the pathophysiology of DPN. In this study, we analyzed the spatiotemporal tight junction protein profile, barrier permeability, and vessel-associated macrophages in Wistar rats with streptozotocin-induced DPN. In these rats, mechanical hypersensitivity developed after 2 weeks and loss of motor function after 8 weeks, while the BNB and the blood-DRG barrier were leakier for small, but not for large molecules after 8 weeks only. The blood-spinal cord barrier remained sealed throughout the observation period. No gross changes in tight junction protein or cytokine expression were observed in all barriers to blood. However, expression of *Cldn1* mRNA in perineurium was specifically downregulated in conjunction with weaker vessel-associated macrophage shielding of the BNB. Our results underline the role of specific tight junction proteins and BNB breakdown in DPN maintenance and differentiate DPN from traumatic nerve injury. Targeting claudins and sealing the BNB could stabilize pain and prevent further nerve damage.

**Key messages:**

• In diabetic painful neuropathy in rats:

• Blood nerve barrier and blood DRG barrier are leaky for micromolecules.

• Perineurial *Cldn1* sealing the blood nerve barrier is specifically downregulated.

• Endoneurial vessel-associated macrophages are also decreased.

• These changes occur after onset of hyperalgesia thereby maintaining rather than inducing pain.

**Graphical abstract:**

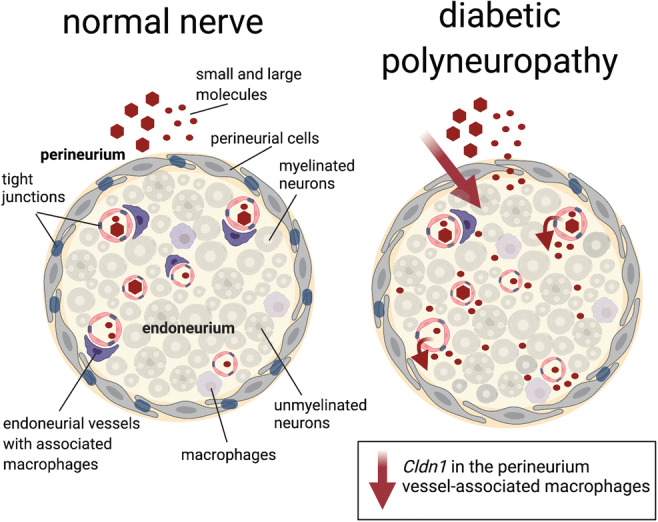

**Supplementary Information:**

The online version contains supplementary material available at 10.1007/s00109-021-02091-1.

## Introduction

Diabetic polyneuropathy (DPN) is the most common diabetes complication. In 10 to 26% of diabetic patients, DPN is painful [1]. Classical analgesics such as non-steroidal analgesics and—with some exception—opioids are not very effective in neuropathic pain. So, treatment still proves difficult necessitating to identify new targets [2]. Lacking analgesia not only implies patient suffering but also a significant socioeconomic burden [3]. In addition, analgesics only relieve pain and have no beneficial impact on DPN natural history. Other than lifestyle modification and diabetes control, no disease-modifying therapies exist.

DPN starts with an axonopathy due to increased polyol flux, free radical and oxidative stress, mitochondrial dysfunction, ischemic or hypoxic microvascular damage, trophic support loss for neurons, and lipid toxicity as well as Schwann cell pathology [4]. In the skin, intraepidermal nerve fibers are lost and nodes of Ranvier become elongated [5], whereas peptidergic fibers increase [6].

The blood-nerve barrier (BNB) consists of the perineurium surrounding nerve fascicles and the endothelium of endoneurial blood vessels [7]. The BNB restricts ions and molecule paracellular flow into the endoneurial milieu. Its perineurial and endoneurial cells are connected by adherens junctions and sealed by tight junctions. In the perineurium, occludin, claudin-1, -3, -19, tricellulin, and TJP1 (ZO-1) are expressed [8–12]. Endoneurial vessels express claudin-5, occludin, and TJP1 [7]. Unlike other neuronal barriers, sealing endoneurial vessels is not only dependent on pericytes but also on vessel-associated macrophages [13]. They recapture molecules leaking through the BNB as there is increased transcytosis in the BNB in comparison to the BBB. Increased barrier permeability like in traumatic nerve injury [14, 15] not only facilitates potentially harmful molecule diffusion into the nerve but also supports immune cell transcellular migration by adhesion molecule upregulation [4]. Tight junction protein downregulation, e.g., *Cldn1* or *Tjp1* [9–12], and junctional adhesion molecules (JAM) or intercellular adhesion molecule upregulation can both contribute to BNB dysfunction [4, 16].

Up to now, it is controversial to which extent BNB, blood-DRG, and blood-spinal cord barriers are affected in preclinical models [17, 18] and in patients [19, 20] and whether their molecular profile contributes to DPN pathophysiology. We hypothesized that in streptozotocin (STZ)-induced DPN, all barriers are leaky and characteristic tight junction proteins such as *Cldn1*, *Cldn5*, and *Tjp1* are downregulated.

## Research design and methods

### Animal model 

Animal protocols (# REG 2-264) have been approved by the animal care committee of the provincial government of Würzburg. Male Wistar rats (Janvier labs, Le Genest-St-Isle, France) weighing 180–250 g, free of pathogenic microorganisms, were housed in groups of six in cages, with enrichment tools, in a circadian light rhythm (12 h/12 h light/dark cycle, 21–25 °C, 45–55% humidity) with food and water ad libitum. Animals were randomly assigned to groups so that test condition and control animals were in different numbers in each cage. Experiments were conducted during daytime and at indicated time points. Handling procedures were in accordance with international guidelines for the care and use of laboratory animals (EU Directive 2010/63/EU for animal experiments). Score sheets with defined end points were used daily to monitor animal well-being.

Two experimental groups were compared at different time points: vehicle and STZ (Sigma chemical) injected rats. STZ condition was established by injecting a 45 mg/kg STZ in 0.1 M citrate buffer pH 4.5 single intravenous dose [21]. Diabetes induction was verified by blood glucose measurement using Glucosmart®. Vehicle rats were injected with 0.1 M citrate buffer pH 4.5.

### Behavioral testing

Mechanical hypersensitivity was determined using the von Frey test [12, 14, 22]. A series of von Frey filaments (Aesthesio® set, UGO BASILE) were assessed to record the hind paw withdrawal threshold and identify mechanical allodynia response and touch sensitivity after STZ injection. In general, the filaments were applied to the hind paw plantar surface and were held for 1–3 s, until the filaments were bent to a 45° angle. Each paw received stimuli from different filament forces, with a 30-s recovery period between each application. The 50% von Frey filament paw withdrawal threshold response was determined using Dixon’s up and down method [23].

Thermal hypersensitivity was assessed performing Hargreaves test (IITC plantar test apparatus model 400 heated base) [24]. A light source was applied on right hind paw. Measurements were performed two times (with 30 s intervals). Averages were calculated subsequently.

Motor performance was studied using rotarod. Rats were placed on turning wheels and their performance time (latency to fall from the wheel) was measured [14, 22].

### Permeability assessment

To assess capillary permeability to small molecules, rats were intravenously injected with sodium fluorescein (NaFlu, 10%; 2 ml/kg; MW, 376 Da; Sigma-Aldrich). After 30 min, the sciatic nerve, DRG, and spinal cord were harvested followed by 1 h postfixation in 4% paraformaldehyde (PFA). In order to study perineurial permeability to small molecules, harvested sciatic nerves were immediately fixed with 4% PFA for 1 h and immersed for 15 min in a 3% NaFlu in saline solution and then placed in sucrose 10% over night prior embedding in Tissue-Tek O.C.T compound [14, 22].

To evaluate the perineurial permeability to macromolecules, harvested sciatic nerves were immediately fixed with 4% PFA for 1 h and immersed in 2 ml of Evans blue albumin (EBA, 5% bovine albumin labeled with 1% Evans blue; both from Sigma Chemicals, 68 kDa) for 1 h [22]. Afterwards, tissues were embedded in Tissue-Tek O.C.T compound (ref. 4583) and cut. Sections of 10 μm obtained were analyzed by microscopy (Keyence BZ-9000).

Endothelial permeability to larger macromolecules was also assessed in sciatic nerves and DRG with immunofluorescence directed against fibrinogen (340 kDa). Frozen sciatic nerves and DRG tissue were cut in sections and fixed with ice cold acetone. Sections were permeabilized with 0.5% Triton-X100 in phosphate-buffered saline and blocked in 1% goat serum in phosphate-buffered saline and 3% bovine serum albumin in phosphate-buffered saline. Then, they were incubated with primary antibody goat anti fibrinogen (1:100 RRID: AB_10787808) and secondary antibody donkey anti goat Alexa fluor 594 (1:600 RRID: AB_2762828). Stained sections were analyzed by microscopy (Keyence BZ-9000).

### Laser microdissection

Sciatic nerves and DRG from male Wistar rats embedded in Tissue-Tek O.C.T compound (ref. 4583) and stored at − 80 °C were sectioned using Leica CM3050S cryostat. Sections of 20 μm were collected on Arcturus® PEN membrane glass slides (applied biosystems ref: LCM0522) treated with RNAse Away spray (Sigma-Aldrich ref: 83931). Sections went through toluidine blue staining before laser microdissectin (LMD). Once the sections were stained, slides were set in Leica LMD6 microscope coupled with a 355 nm laser. Using the Leica LMD V7.6 software, different tissue areas were selected and dissected. In the sciatic nerve, capillaries and perineurium were dissected. In DRGs, capillaries and the fiber-rich region were dissected. Furthermore, the entire neuron-rich region and the perineurium-like area were dissected. Samples were collected in 0.2-ml PCR soft tubes (Biozym 711080) and stored at − 80 °C.

### Reverse transcription quantitative PCR

Entire sciatic nerve, DRG, and spinal cord were harvested and dissected. Total RNA was extracted with Trizol (Invitrogen) or the miRNeasy kit. Next, 1 μg was transcribed to cDNA using the high capacity cDNA-kit (Applied Biosystems, Life technologies) following manufacturer’s instructions. *Gapdh* was used as a reference gene for quantification. RTqPCR analysis was performed using the following primers (MWG Eurofins, Ebersberg, Germany) with the SYBRGreen method:

*Cldn1* (fw GGGACAACATCGTGACTGCT and rev CCACTAATGTCGCCAGACCTG), *Cldn5* (fw AAATTCTGGGTCTGGTGCTG and rev GCCGGTCAAGGTAACAAAGA), *Cldn12* (fw AACTGGCCAAGTGTCTGGTC and rev AGACCCCCTGAGCTAGCAAT), *Cldn19* (fw TGCTGAAGGACCCATCTG and rev TGTGCTTGCTGTGAGAACTG), *Jam3* (fw TGCTGCTCTTCAGGGGCTGCGTGAT and rev AACACATCTGTGCGACCGGCCAGGT), *Ocln* (fw TGGGCAGTCGGGTTGACT and rev GGGCATCATGGTGTTCATTG), *Tric* (fw TGAATGGCCACCAGTGACCGA and rev AGTCAGGCATTACGATGGGCTTAG), *Tjp1* (fw CACGATGCTCAGAGACGAAGG and rev TTCTACATATGGAAGTTGGGGATC), *Tnfa* (fw ACCACGCTCTTCTGTCTACTG and rev CTTGGTGGTTTGCTACGAC), *Il10* (fw AGACCCACATGCTCCGAGAG and rev GGGCATCACTTCTACCAGGT), *Il6* (fw CTGCTCTGGTCTTCTGGAGT and rev TGGAAGTTGGGGTAGGAAGG), and *Gapdh* (fw AGTCTACTGGCGTCTTCAC and rev TCATATTTCTCGTGGTTCAC). RTqPCR analysis was carried out by a 7300 Real-Time PCR System (Applied Biosystems, Thermo Fischer) with the following program: 50 °C for 2 min, 95 °C for 2 min, and 40 cycles at 95 °C for 3 s and 60 °C for 30 s. Samples were analyzed as triplicates. Relative mRNA abundances to *Gapdh* were calculated by the ΔΔCt method (threshold cycle value). To compare different samples, the control condition (vehicle animals) was set at 1.

Following LMD, total RNA was extracted using RNeasy® Micro Kit (Quiagen cat. no. 74004) Total RNA (1 μg) was transcribed to cDNA using the high-capacity cDNA kit (Applied Biosystems Life technologies) following the manufacturer’s instructions. QPCR analysis was performed with the following primers using the Taqman method: *Cldn1* (Thermo Fisher cat# Rn00581740_m1), *Cldn5* (Thermo Fisher cat# Rn01753146_s1), von Willebrand Factor (*Vwf*; Thermo Fisher cat# Rn01492158_m1), *Tjp1* (Thermo Fisher cat# Rn02116071_s1), and *Gapdh* (Thermo Fisher cat# Rn01462662_g1). QPCR analysis was carried out using the StepOnePlus Real-Time PCR System (Applied Biosystems) with the following program: fast 40 min for a run, 95 °C for 20 s, and 45 cycles at 95 °C for 1 s and 60 °C for 20 s. Samples were analyzed as triplicates. Target cDNAs were normalized to endogenous control (*Gapdh*).

### Immunofluorescence and fluorescence microscopy

Frozen sciatic nerves and DRG tissues were sectioned and fixed with ice-cold acetone. Sections were permeabilized with 0.5% Triton-X100 in phosphate-buffered saline (PBS) and blocked in 1% goat serum in PBS and 3% bovine serum albumin in PBS. Then, they were incubated with primary antibodies mouse anti human, rat, canine claudin-1 (1:100; RRID AB_2533323), mouse anti human, rat-claudin-5 (1:100; RRID AB_2533200), rabbit anti-von Willebrand factor (vWF) (1:100; RRID:AB_2315602), mouse anti rat CD68 (RRID:AB_2291300), mouse anti rat RECA-1 (1:100; RRID:AB_935279), and rabbit anti rat CD206 (1:100; RRID:AB_1523910**)** overnight, following sequential incubation with secondary antibodies: donkey anti-mouse Alexa Fluor 555 (1:600; RRID:AB_2536180), goat anti-mouse FITC (1:600; RRID:AB_259378), donkey anti-rabbit Alexa Fluor 555 (1:600; RRID:AB_162543), and donkey anti-rabbit Alexa Fluor 488 (1:600, RRID:AB_2535792). DAPI 2 mg/ml in distilled water (Ref D9542, Sigma Aldrich) was used to stain nuclei. ProTag Mount-Fluor was employed for mounting the sections (Biocyc, Luckenwalde, Germany), and fluorescence microscopy was performed (Olympus BX51, camera Olympus DpP71).

### Western blot

Eight weeks after injection of either vehicle solution or STZ sciatic nerves were harvested and homogenized in RIPA lysis buffer (50 mM Tris pH 8.0; 150 mM NaCl; 0.1% SDS; 0.5% Na-deoxycholate; 1% NP40) with proteinase inhibitor (Complete, Roche Applied Science) and placed in tissue lyser for 5 min at maximal frequency of 30. Homogenates were then centrifuged at 450*g* for 10 min at 4 °C. Supernatants were then centrifuged for 60 min at 20,000*g* at 4 °C to precipitate the membrane fraction. The cytosolic fraction in the supernatant was transferred to another tube and the precipitated membrane fraction dissolved in RIPA lysis buffer with proteinase inhibitors. Proteins were quantified with BCA protein assay reagent (Pierce, Rockford, IL, USA). Aliquots of protein were mixed with sodium dodecyl sulfate (SDS) containing buffer (Laemmli), denatured at 60 °C for 5 min, fractionated on SDS polyacrylamide gels, and subsequently blotted onto nitrocellulose membranes (Amersham™ catalogue number: 10500001). Proteins were detected using specific antibodies: mouse anti claudin-5 1:200; RRID AB_2533200, mouse IgG HRP conjugated 1:3000 RRID: AB_330924 and—as protein loading control—β-actin HRP conjugated, 1:20,000 RRID: AB_262011. For quantification, claudin-5 band intensities were normalized to their β-actin loading control band.

### Image analysis

Bioimages were analyzed using FIJI (ImageJ) software. For each image, regions of interest (ROI) were segmented manually and the fluorescence intensity (mean gray value) was measured in corresponding ROIs. Then, we subtracted the background to the measured intensities; the results obtained correspond to the actual signal intensity. Per animal, five to six images were analyzed, from which an individual mean fluorescence intensity was calculated per animal. To quantify claudin-5 specifically in capillaries, sections were co-stained for von Willebrand factor (vWF). After quantifying both claudin-5 and vWF following the above described method and assuring that there were no changes in vWF expression, we normalized claudin-5 fluorescence intensity on vWF fluorescence intensity. In order to quantify vessel-associated macrophages, sections were co-stained either for CD68 and vWF or CD206 and RECA-1. The total number of macrophages (CD68+ or CD206+) near blood vessels (vWF+ or RECA-1+) was counted and expressed relative to the total number of capillaries in the endoneurium.

### Statistical analysis

Data are presented as mean ± SEM and medians and interquartile range. Two groups were compared by *t* test for independent variables or Mann-Whitney non-parametric test. Multiple measurements with one or two variables (e.g., time and treatment) were analyzed by one- or two-way ANOVA or by repeated measurements (RM), two-way ANOVA, and Friedman’s nonparametric test, as appropriate and described in the figure legends. Differences were considered significant if *p* < 0.05.

## Results

### Mechanical hypersensitivity and motor impairment in diabetic Wistar rats

**Fig. 1:**
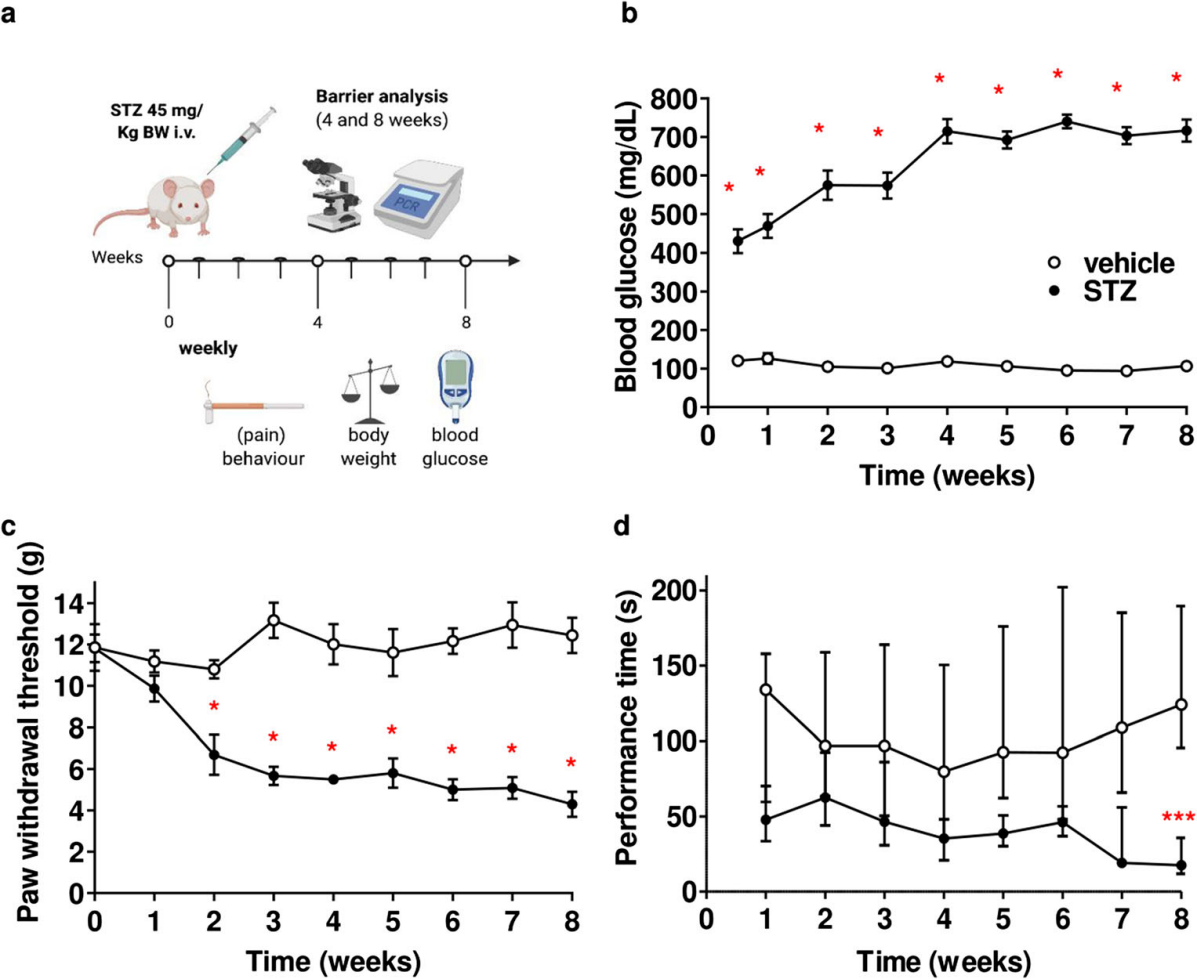
Mechanical hypersensitivity and impairment of motor performance in STZ-induced diabetes type I in rats. **a** Male Wistar rats were treated with i.v. streptozotocin (STZ; once 45 mg/kg): experimental outline for tests and tissue harvest (created with biorender.com). **b** Blood glucose in mg/dl, **c** mechanical allodynia measured by von Frey hairs (g), and **d** motor function by rotarod (s). All data points represent mean ± SEM. *P < 0.05 (*n* = 6). Two-way repeated measurement ANOVA and Bonferroni post hoc test for multiple comparisons. For rotarod, **d** data points represent median and interquartile range. ***P < 0.001 (*n* = 6); Friedman test and Dunn’s post hoc tests for multiple comparison

To validate the model, we measured blood glucose and observed a four-times increased level for all STZ-injected rats 1 week after injection and a seven-times higher level 4 to 8 weeks after injection (Fig. 1a, b). We confirmed mechanical hypersensitivity starting at 2 weeks after STZ by von Frey hair test (Fig. 1c) but did not detect thermal hypersensitivity in the Hargreaves test (Suppl. Fig. 1) in line with the previous findings [25]. Performance time on the rotarod, a measure of motor function, was impaired in STZ-treated rats (Fig. 1d).

### Increased capillary permeability to small molecules, but normal tight junction protein expression in DRG

**Fig. 2:**
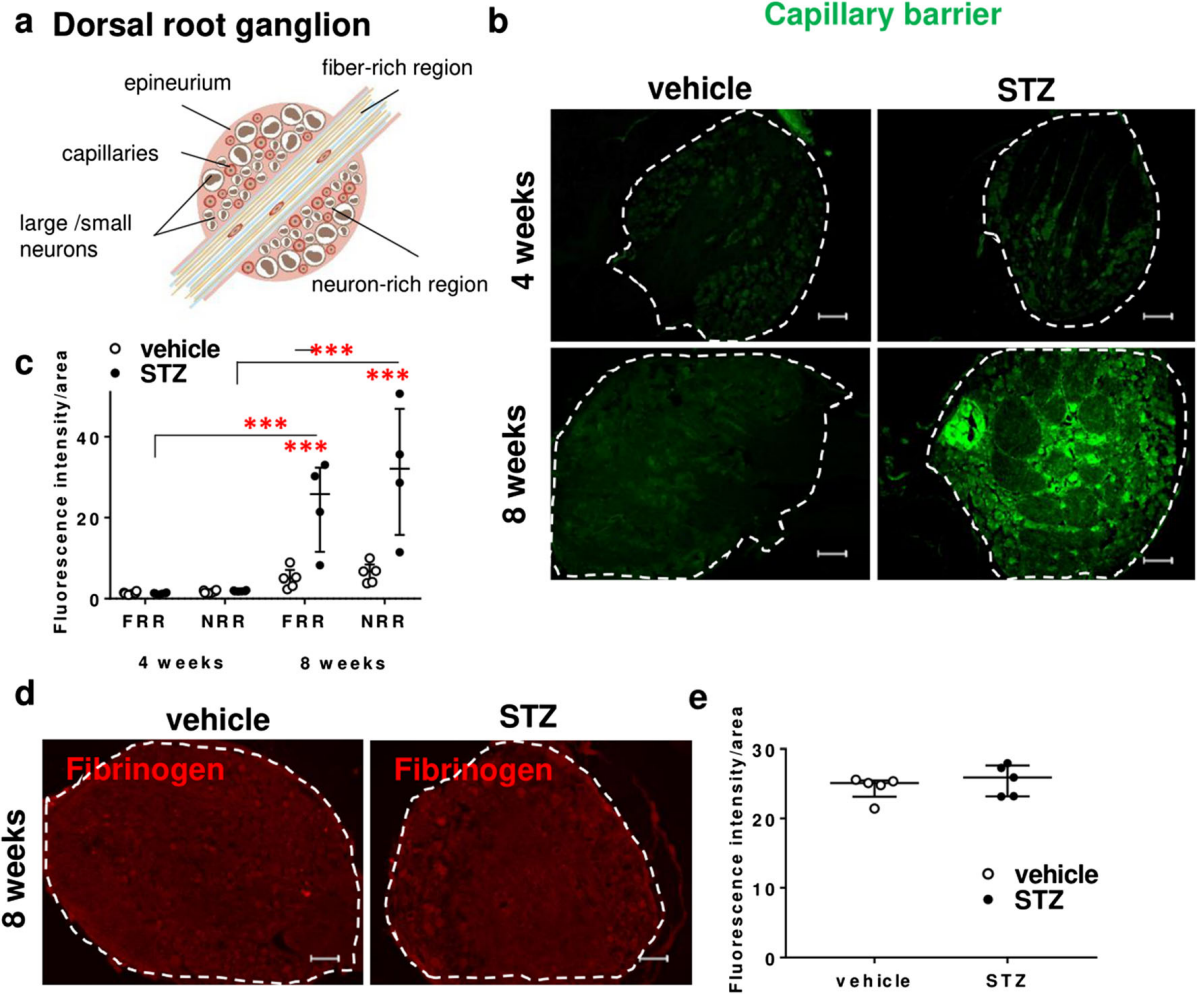
Increased selective capillary permeability to small molecules in dorsal root ganglia. **a** Diagram of the DRG with neuron-rich region (NRR) and fiber-rich region (FRR). **b**, **c** Male Wistar rats were i.v. injected with sodium fluorescein 30 min before sacrifice. Fluorescence was quantified in the neuron-rich region and fiber-rich region of the DRG, *n* = 4–5. Two-way ANOVA and Bonferroni post hoc test for multiple comparisons; ***P < 0.001. **d**, **e** Fibrinogen immunostaining and respective quantification in the dorsal root ganglion, *n* = 6

After NaFlu (376 Da) injection, fluorescence increased in diabetic rat neuron-rich region and fiber-rich region of DRG at 8 weeks indicating increased capillary permeability for small molecules (Fig. 2a–c). At 8 weeks, we could not observe that fibrinogen—a blood-borne large molecule (340 kDa)—increased immunoreactivity (Fig. 2d, e).

**Fig. 3:**
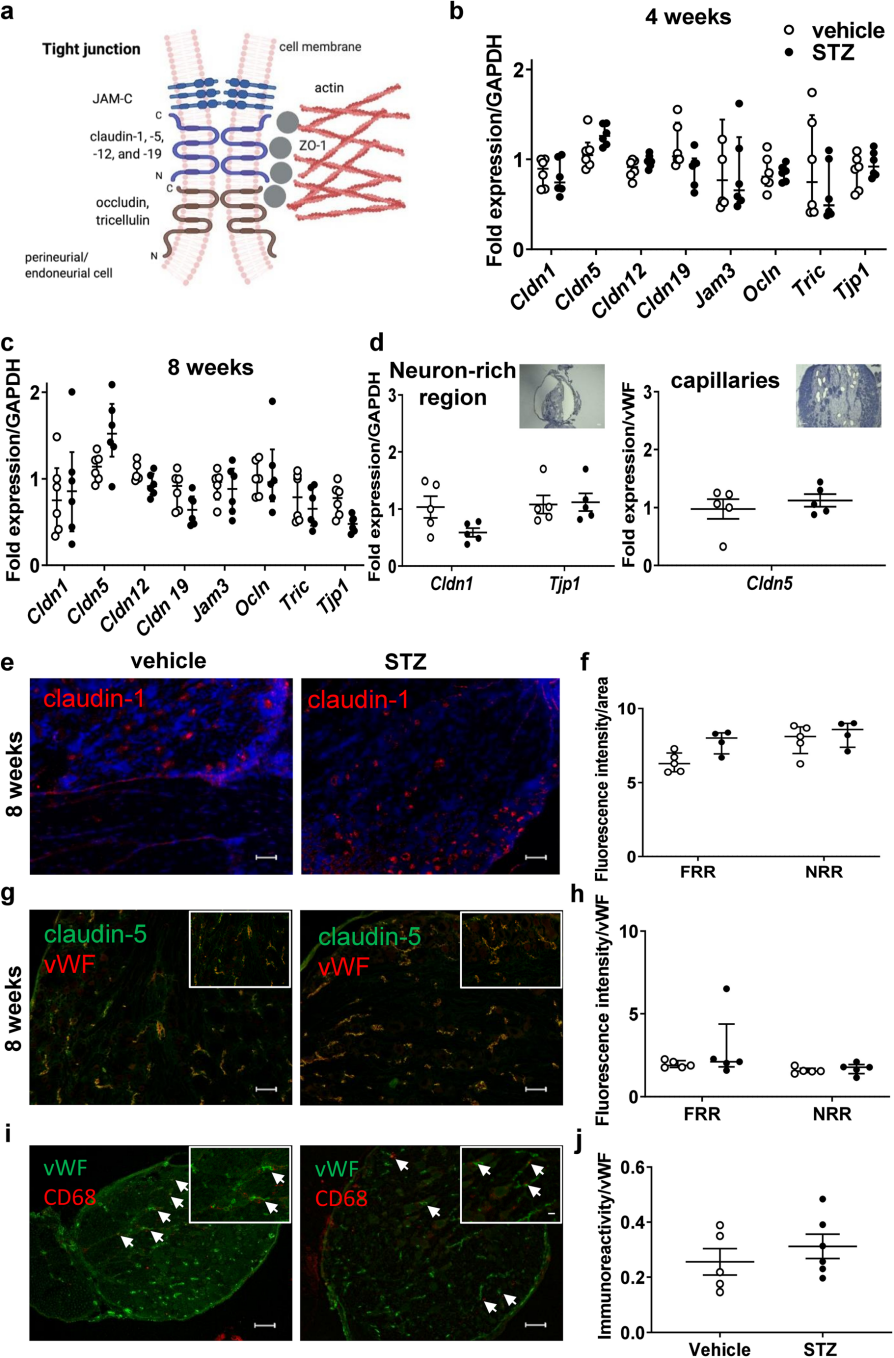
Unchanged tight junction protein expression in dorsal root ganglia in diabetic rats. MRNA was extracted from entire DRG of male Wistar rats treated with STZ i.v. or vehicle. **a** Schematic of the tight junction and its proteins (created with biorender.com). **b**, **c** Relative tight junction protein mRNA expression in DRG: *Cldn1*, *Cldn5*, *Cldn12*, *Cldn19*, *Jam3*, *Ocln*, *Tric*, and *Tjp1* (ZO-1) in entire DRG. **d** Relative mRNA expression of *Cldn1*, *Tjp1* in the neuron-rich region and *Cldn5* in capillaries separated with laser microdissection (*n* = 6), Mann-Whitney for comparison. **e–h** Staining for claudin-1 or claudin-5 and von Willebrand factor and quantification in selected areas (*n* = 5–6). **i**, **j** Staining for CD68 and von Willebrand factor and quantification (*n* = 5–6), arrows depict examples. Scale bars = 100 μm and 20 μm in the insert, dot plots represent median and interquartile range, Mann-Whitney test

*Cldn1* and *Cldn5* seal endothelia, *Cldn12* and *Cldn19* protect the perineurial and myelin barrier, *Tric* and—in part—*Ocln* are important in shielding tricellular junctions. *Jam3* allows for cellular adhesion and *Tjp1* anchors tight junction proteins with the intracellular cytoskeleton (Fig. 3a) [7]. In whole DRGs, unaltered RNA expression level of these tight junction proteins was observed at 4 and 8 weeks (Fig. 3b, c). We then hypothesized that changes could be more selective in particular DRG areas. Therefore, we used LMD in order to separately analyze expression levels of corresponding barrier proteins in the DRG neuron-rich and fiber-rich region [15]. However, *Cldn1* and *Tjp1* expression levels in the neuron-rich region and *Cldn5* in capillaries were unchanged 8 weeks after STZ (Fig. 3d). Likewise, claudin-1 and claudin-5 immunoreactivity in vWF-positive vessels and vWF-associated CD68+ macrophages were unaffected (Fig. 3e–j and Suppl. Fig. 2a for full size insert of CD68/vWF). In summary, 8 weeks after STZ-DPN induction, the DRG is more permeable for small molecules, but not for macromolecules, and this effect is independent of tight junction protein expression level.

After traumatic nerve injury, the blood-spinal cord barrier is leakier and *Cldn5* and *Tjp1* are downregulated [14]. In DPN, endothelial permeability to small molecules as well as tight junction protein expression were unchanged in STZ-treated animals (Suppl. Fig. 3). Thus, the blood-spinal cord barrier is intact.

### Capillary, perineurial leakiness, and perineurial claudin-1 loss in STZ-induced DPN

**Fig. 4:**
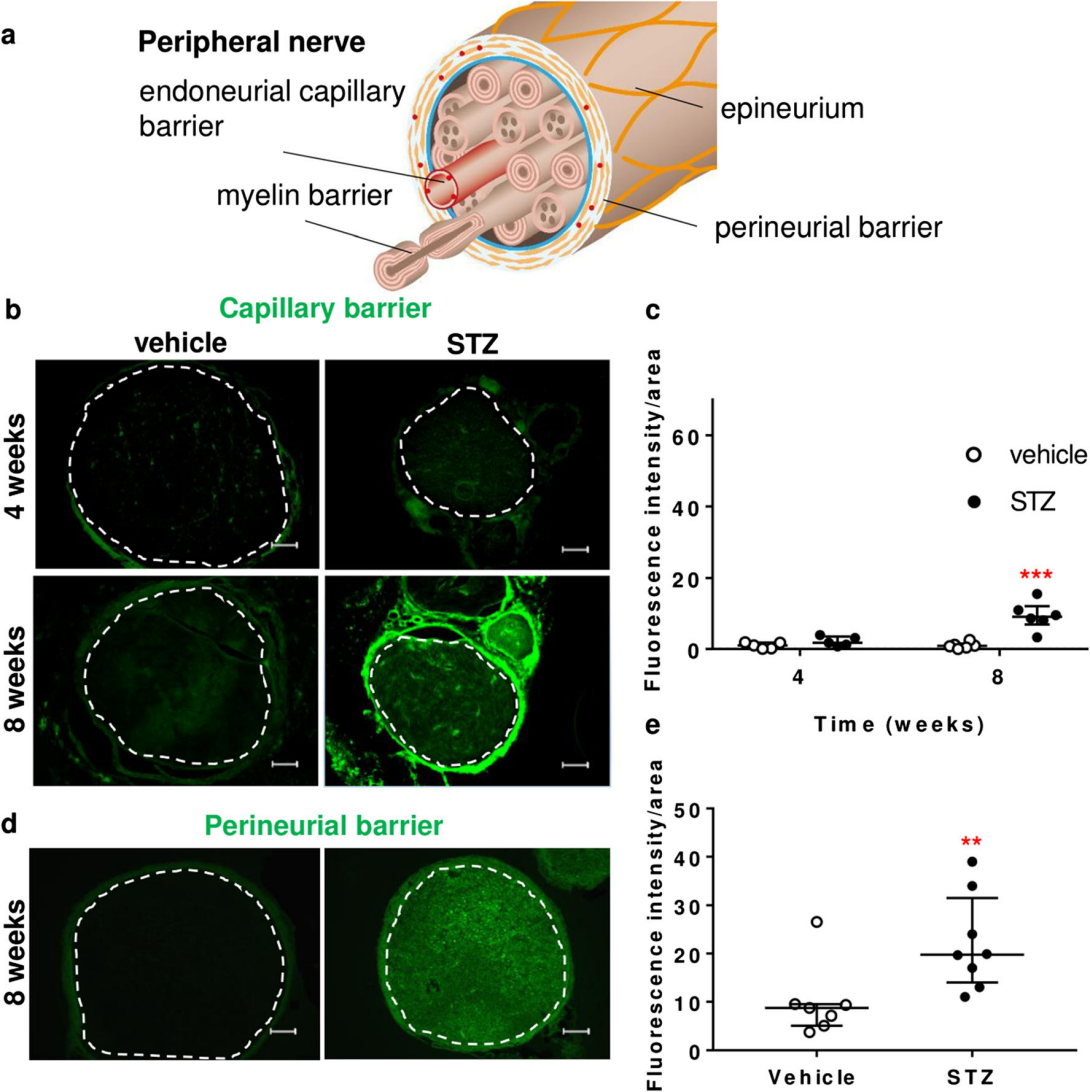
Increased capillary and perineurial permeability for small molecules in prolonged STZ-induced DPN. **a** Barriers in the peripheral nerve. **b**, **c** Diabetic and non-diabetic male Wistar rats were treated with i.v. sodium fluorescein (376 Da) 30 min before sacrifice to measure the tightness of the capillary barrier, *n* = 5–6. **d**, **e** Sciatic nerves were collected and immersed in 3% sodium fluorescein to analyze the perineurial barrier tightness, *n* = 7–8. Quantification of fluorescence intensity normalized by the stained area (dashed line) is depicted in **c** and **e**. Scale bars = 100 μm, dot plots represent median and interquartile range (**c**). two-way ANOVA, ***P < 0.001 (**e**). Mann-Whitney test, **P < 0.01

**Fig. 5:**
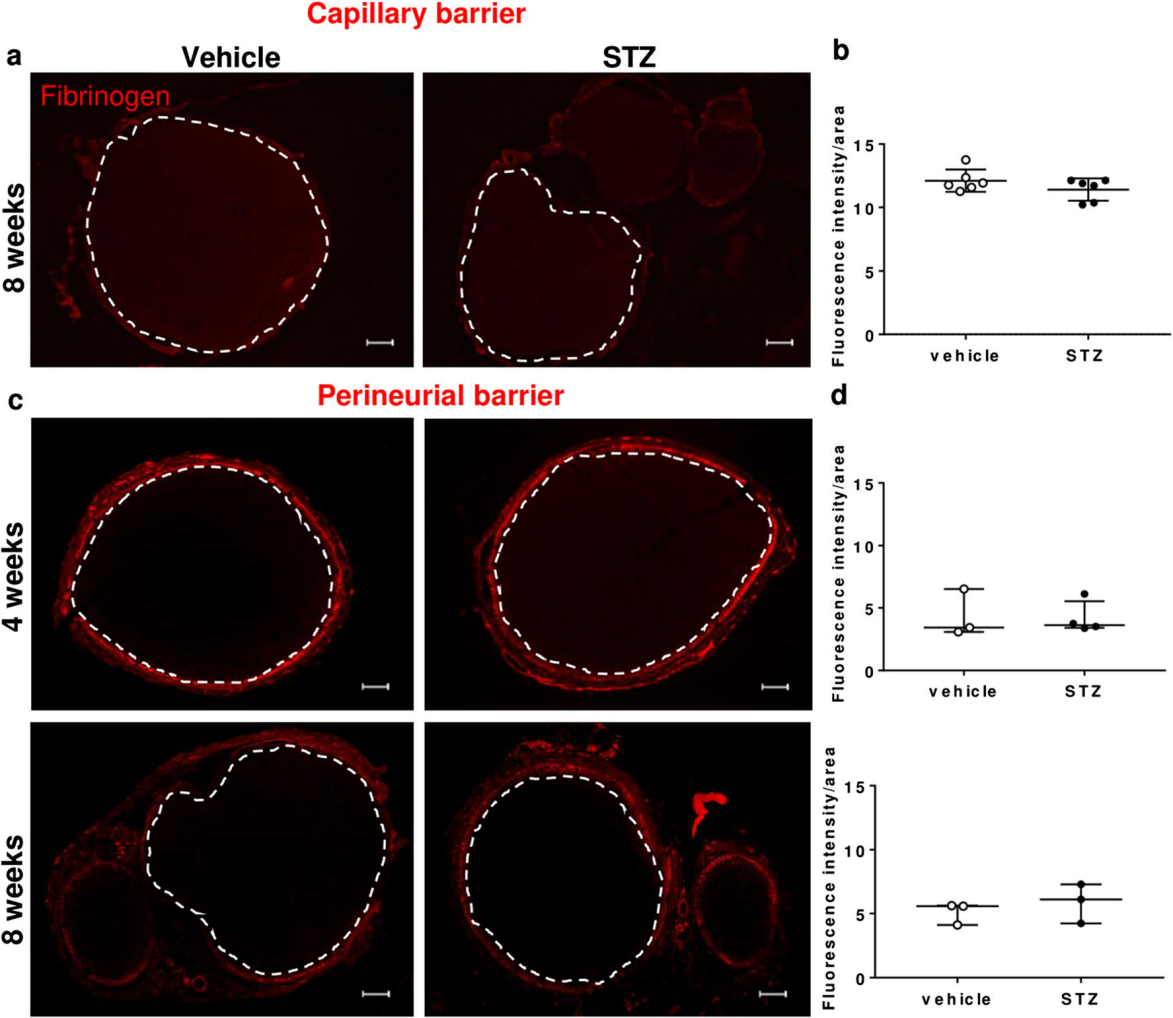
Preserved blood-nerve barrier function for macromolecules in the sciatic nerve. Sciatic nerve from male Wistar rats was harvested and **a**, **b** stained against fibrinogen (340 kDa) or **c**, **d** incubated in Evans blue albumin (68 kDa) for 1 h. **a** Endoneurial immunoreactivity against fibrinogen 8 weeks after STZ or vehicle, *n* = 6. **c** Penetration of Evans blue albumin into the endoneurium 4 and 8 weeks after STZ, *n* = 3. Fluorescence intensity was quantified and normalized to the region of interest (dashed line). Scale bars = 100 μm

Fluorescence in sciatic nerve endoneurium was enhanced after in vivo (capillary barrier, Fig. 4a–c) and ex vivo (perineurial barrier, Fig. 4d, e) NaFlu application in STZ rats at 8 weeks—indicating capillary and perineurial barrier leakiness. No permeability changes were observed when nerves were stained for fibrinogen (340 kDa, capillary barrier) (Fig. 5a, b). Likewise, sciatic nerve incubation with EBA (68 kDa) demonstrated no endoneurial diffusion (perineurial barrier, Fig. 5c, d)

**Fig. 6:**
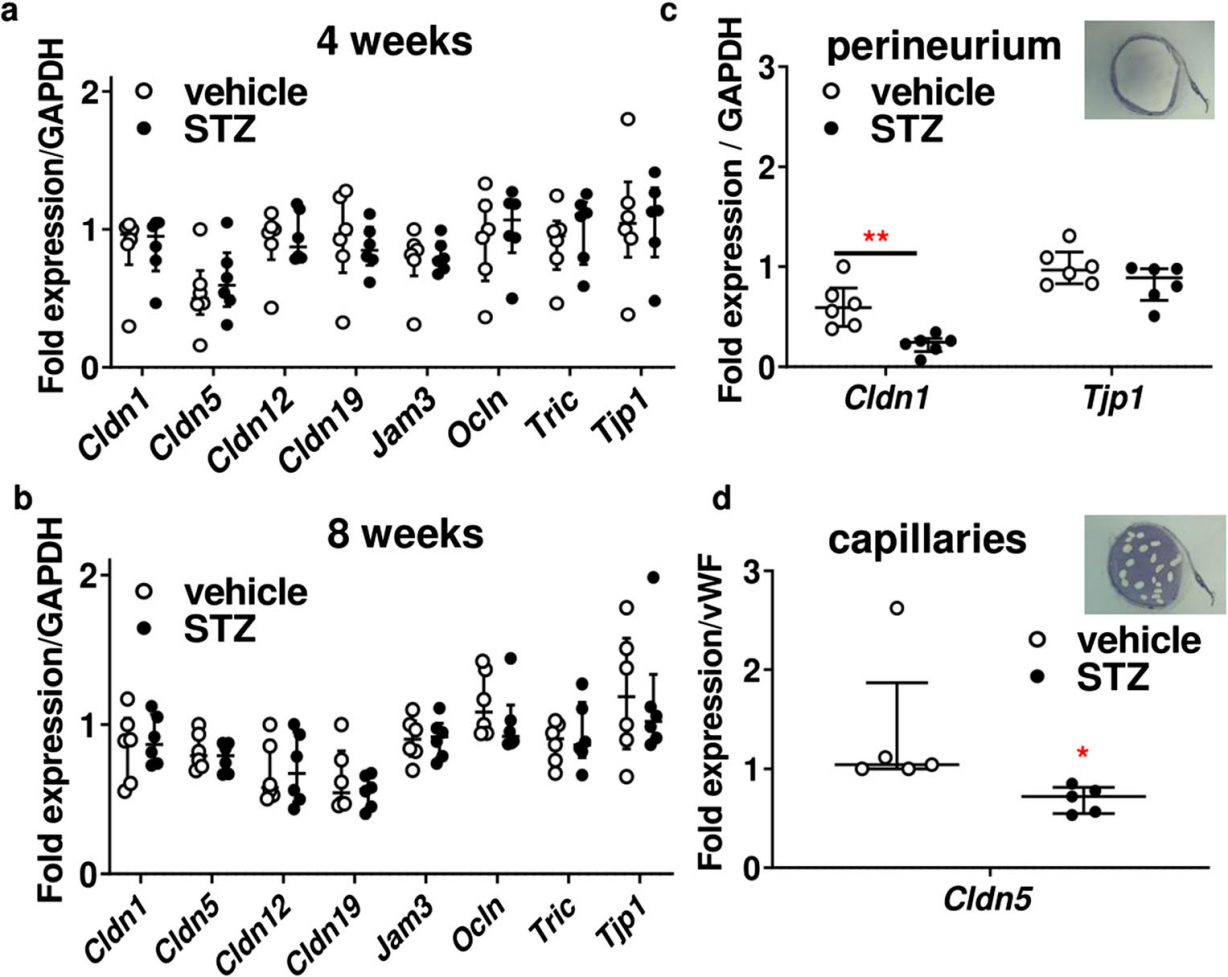
Decreased *Cldn1* and *Cldn5* mRNA in perineurium and capillaries, respectively. **a–d** MRNA expression of tight junction proteins of male Wistar rats treated or not with STZ i.v. injection **a**, **b** either form the whole sciatic nerve (*n* = 6) or **c**, **d** after tissue dissection with laser-assisted microdissection—as depicted in the inserts. MRNA expression of claudin 1, 5, 12, and 19 (*Cldn1*, *Cldn5*, *Cldn12*, *Cldn19*), junctional adhesion molecule C (JAM-C, *Jam3*), *occludin* (*Ocln*), *tricellulin* (*Tric*), and zona occludens-1 (ZO-1, *Tjp1*) was calculated relative to GAPDH. Dot plots represent medians and interquartile range. All *n* = 5-6, **P < 0.01 and *P < 0.05. Mann-Whitney test (**a–c**) and unpaired *t* test (**d**)

In addition, in whole nerve-isolated RNA samples, the expression of a panel of tight junction proteins was unaltered (Fig. 6a, b). When mRNA expression was analyzed in perineurium-microdissected tissue samples and from the capillaries, a perineurial *Cldn1* and an endoneurial *Cldn5* downregulation (Fig. 6c, d) were seen.

**Fig. 7:**
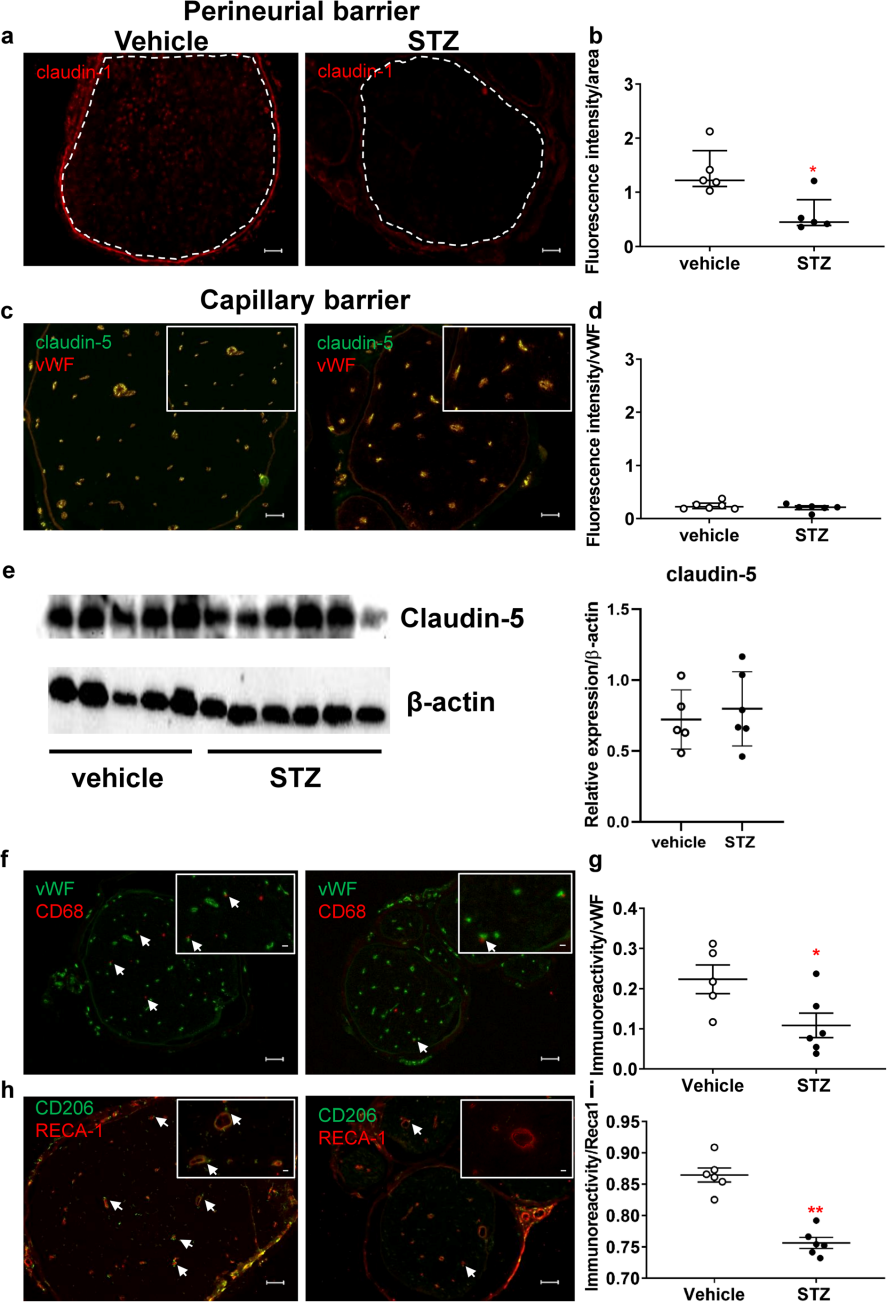
Decreased claudin-1 immunoreactivity in the perineurium and vessel-associated macrophages in prolonged STZ DPN. Sciatic nerve from male Wistar rats was stained with anti-claudin-1 (**a**, **b**), anti-claudin-5 + anti-von Willebrand factor (vWF, **c**, **d**). Claudin-5 was quantified relative to β-actin by Western blot (**e**). Vessel-associated macrophages were identified by immunolabeling with anti-CD68 and anti-vWF (**f**, **g**) or anti-CD206 and RECA-1 (**h**, **i**). Fluorescence intensity was quantified in the regions of interest (perineurium; **a**, **b**) and (vWF+ vessels; **c**, **d**) normalized to the area. **f**, **g**, **h**, **i** The total number of macrophages (CD68+ or CD206+) in proximity to blood vessels (vWF+ or RECA-1+) was counted and expressed relative to the total number of capillaries in the endoneurium. *n* = 5–6. Arrows depict examples. Scale bars = 100 μm and in the insert 20 μm (**f**, **g**) and 10 μm (**h**, **i**), dot plots represent medians and interquartile range, Mann-Whitney test for comparison. *P < 0.05 and **P < 0.01

To visualize the corresponding proteins, claudin-1 and -5 immunoreactivity was quantified in these areas. This showed reduced perineurial claudin-1 immunoreactivity at 8-week STZ-induced DPN (Fig. 7a, b). Endoneurial claudin-5 immunoreactivity in the vWF-positive capillaries was similar in both groups (Fig. 7c, d) and this is confirmed by claudin-5 Western blot showing no difference between groups (Fig. 7e).

### No signs of inflammation in STZ-induced diabetic neuropathy, but decreased vessel-associated-macrophage number

Cytokines like TNFα are known to regulate tight junction protein expression [26]. We therefore selected interleukin-6 and TNFα as pro-inflammatory cytokines and interleukin-10 as an anti-inflammatory cytokine (Suppl. Fig. 4). *Il6*, *Il10*, and *Tnfa* expression was unaffected in the sciatic nerve, both, at 4 and 8 weeks after STZ injection. Likewise, there was no increased CD68+ macrophage infiltration observable in the sciatic nerve.

The barrier composition of specifically endoneurial capillaries is different from the blood-brain barrier, because not only pericytes but also macrophages (vessel-associated macrophages) prevent large molecule penetration [13].

In the sciatic nerve of STZ-treated rats, significantly less CD68+ macrophages were associated to vWF+ capillaries in comparison to vehicle control (Fig. 7f, g and Suppl. Fig. 2b for full size insert). Vessel-associated macrophages could be further classified as CD206+ anti-inflammatory M2 macrophages [27] adjacent to RECA-1 positive endoneurial capillaries [28]. The number of CD206+ vessel-associated macrophages was reduced 8 weeks after STZ injection (Fig. 7h, i and Suppl. Fig. 2c for full size insert). Thus, less vessel-associated macrophages and not lower tight junction protein expression are observed at the time point of capillary leakiness in STZ-induced diabetes.

## Discussion

In the present study, we provide evidence for small molecules BNB leakiness in STZ-induced DPN: decreased perineurial claudin-1 expression explains a higher perineurial permeability, while a decreased vessel-associated M2 macrophages could be responsible for enhanced endothelial diffusion. The blood-DRG barrier, but not the blood-spinal cord barrier, was also leakier for small molecules. BNB, blood-DRG, and blood-spinal cord barrier remained sealed for larger molecules. No signs of neuroinflammation, e.g., increased macrophage infiltration or proinflammatory cytokines, were detected. Moreover, all observed barrier changes occurred between 4 and 8 weeks—long after the first detection of mechanical hypersensitivity. Consequently, a destabilized BNB could be responsible for maintaining—rather than initiating—neuropathic pain as well as further nerve damage.

Previous studies have documented increased peripheral nerve permeability for small but not macromolecules in STZ-induced diabetes after 9 months [17]. Additionally, endoneurial edema is an early sign of STZ-induced diabetes [29], but not in all studies and models [30]. Therefore, our results are in accordance with previous smaller studies. Increased perineurial permeability can be explained by a local loss of *Clnd1*—a major sealing tight junction protein of the perineurium. Macromolecular leakiness has been observed in type 1 diabetes rat models [31] and type 2 diabetes [18], but not other studies [4]. Longer disease duration in type 2 diabetes could result in increased leakiness to larger molecules.

Microvascular changes in DPN include degradation of paracellular tight junction, loss of pericytes, increased basement membrane thickness, and fibrin positive blood vessels as well as endothelial cell hyperplasia [4]. The vascular tight junction protein *Cldn5* is an essential protein in the blood-brain barrier [32]. *Cldn5* mRNA, but not claudin-5 protein, was decreased in the endoneurium in STZ-induced DPN. It is possible that reduced *Cldn5* mRNA results in decreased claudin-5 protein at later time points. But, the decreased number of blood-vessel-associated macrophages in endoneurium could explain the increased small molecule endothelial permeability [13] in synergy with pericyte activity and loss [4, 16].

In the DRG in STZ-induced DPN, a leakiness to small molecules was observed but unchanged tight junction proteins. Increased leakiness could be due to, e.g., increased transcytosis or changes in gap junction proteins [33]. Since our experimental design covered only 8 weeks, tight junction proteins could be affected at later time points.

No changes were seen in the blood-spinal cord barrier permeability in our study. We did not examine the blood-brain barrier, but changes in diabetes have been reported: *Cldn5* and pericyte coverage are decreased in old diabetic mice [34] and claudin-5 forms larger gaps in brain capillaries after 8 weeks STZ-induced diabetes together with cognitive impairment [35] So, several barriers are affected in diabetes, but the role of specific tight junction proteins like claudin-5 seems to depend on anatomical location as well as duration and type of diabetes.

Blood-DRG and blood-spinal cord barrier permeability alterations seem to crucially depend on the type of neuropathy: the blood-spinal cord barrier is leaky after nerve injury [14] and chemotherapy-induced neuropathy [36] but unchanged in DPN—as shown here. Blood-DRG barrier permeability is increased in DPN, but not after traumatic nerve injury [15].

At the moment, it is unclear whether other barriers in the nerve itself might be affected in DPN. Claudin-19 is the major myelin barrier sealing protein. Recent studies support a role of Schwann cells in diabetes [37]. In fact, the myelin sheath lipid composition is altered in STZ-induced DPN [38]. Therefore, studying the myelin barrier in more detail might unravel new pathologies in DPN.

Mechanical allodynia occurred already 2 weeks after STZ injection prior to the increase in permeability. This suggests that at least during the early stages of diabetes, allodynia is caused by other (axonal) factors. Short-term opening of the BNB using claudin-1 interference peptides, tissue plasminogen activator via the low-density receptor protein-1 pathway or hypertonic saline leave nociceptive thresholds unaltered [12, 22, 24]. Indeed, in addition to a leaky barrier, an “insult” like systemic proinflammatory cytokine profile or other proalgesic mediators like methylglyoxal is needed in DPN [39–41]. This is also observed when the inflammatory milieu in traumatic nerve injury affects barrier functions [9–12]. Mechanistically, increased perineurial iNOS expression and alterations in gap junction proteins could further contribute to BNB destabilization in addition to reduced claudin-1 [33].

*Cldn1* and *Cldn5* in the nerve have been extensively studied in traumatic nerve injury. So, what could be the mechanisms underlying reduced *Cldn1* in the perineurium and *Cldn5* in microvessels as well as loss of vessel-associated macrophages in the endoneurium impairing the BNB function? *Cldn1* is regulated by the wnt pathway [22], metalloproteinases, and low-density lipoprotein receptor-related protein-1 [42], as well as microRNA (miR)-155 [12], miR-21, and miR-183 [43]. *Cldn5* and *Tjp1* are regulated by the sonic hedgehog pathway in Lepr^db/db^ type 2 diabetic mice and after nerve injury [10, 18]. Moreover, restoration of blood vessel-associated macrophages could reinstate endothelial barrier function. As activating the sonic hedgehog pathway restores endothelial barrier function in diabetic neuropathy [44], thus, not only tight junction proteins, but also recruitment of macrophages and pericytes could be regulated by common pathways. In summary, treatment with, e.g., glycogen-synthase-kinase-3β inhibitors [22, 45], miR-155 antagomirs [46], or hedgehog fusion proteins [47] could promote BNB sealing in DPN. Some of these inhibitors are currently under investigation for diabetes treatment [15, 47, 48].

DPN is not usually categorized as an immune-mediated neuropathy; however, microvascular changes could be induced by the inflammatory cascades. In our study, we did not observe any increased macrophage infiltration 8 weeks after injection in line with previous studies [49]. Macrophage infiltration is reduced 2–3 weeks in the sciatic nerve after STZ application in mice but return to normal after 13 weeks [50]. Depleting macrophages after STZ injection delayed neuropathic pain onset [51]. Besides their harmful role in pain pathways, macrophages are also known for their beneficial properties following nerve injury. In our study, we found a vessel-associated macrophage loss. In *Dhh*^−/−^ mice—which also have leaky endoneurial capillaries—M2 macrophages produce vascular endothelial growth factor-A [18]. Macrophage depletion normalized capillary density and lumen. Since neither leakiness in *Dhh*^−/−^ nor macrophages/neuroinflammation in diabetic *Lepr*^db/db^ were evaluated, the role of macrophages for barrier stability was not clarified. Perivascular macrophages are found in many organs and play an important role: they maintain tight junctions, limit capillary permeability and inappropriate inflammation and phagocytose material in the brain, kidney, heart, lung, skin, etc. [52]. Malong and colleagues have shown that vessel-associated macrophages are necessary in order to preserve blood-nerve barrier integrity: tamoxifen-treated P0-RafTR mice have a leaky capillary barrier with normal tight junction protein expression but enhanced transcytosis and reduced numbers of vessel-associated macrophages [13]. This points to a novel beneficial rather than harmful macrophage role at the BNB, which might depend on the macrophage subtype, anatomical location, and disease state. For example, anti-inflammatory or vessel-associated macrophages could promote pain resolution.

Barrier leakiness in STZ-induced diabetes is selective for small molecules potentially facilitating edema formation and proalgesic mediator leakage (summarized in [53]). In diabetic patients, this could explain previous findings: (1) nerve edema observed, e.g., by T2 mapping in MRI neurography in patients with DPN [54], (2) altered tight junctions in DPN [19], and (3) altered BNB permeability to blood-borne proteins like glycosylated albumin [55]. Most studies were conducted in type 2 diabetes necessitating more studies in type 2 diabetes models or type 1 diabetic patients. In summary, our data argue that small molecule BNB leakiness is responsible for DPN perpetuation and further nerve destruction rather than an inciting event. This is in sharp contrast to very early BNB changes in nerve injury where barrier leakiness to both small and macro-molecules increases and precedes hypersensitivity [12].

## Supplementary information

ESM 1 (DOCX 3.24 mb)

## Data Availability

The data supporting the findings of this study are available upon request to the authors.

## Abbreviations

BNB: Blood-nerve barrier
DPN: Diabetic polyneuropathy
EBA: Evans blue albumin
LMD: Laser-assisted microdissection
NaFlu: Sodium fluorescein
STZ: Streptozotocin
TJP1: Tight junction protein 1
ZO-1: Zonula occludens protein 1
