# Supplementary material for: Selective blood-nerve barrier leakiness with claudin-1 and vessel-associated macrophage loss in diabetic polyneuropathy
Source: J Mol Med (Berl). 2021 May 21;99(9):1237–50. doi: 10.1007/s00109-021-02091-1 (PMC8367905; doi:10.1007/s00109-021-02091-1)
Supplement: Supplementary file 1 — (DOCX 3.24 mb) [file 109_2021_2091_MOESM1_ESM.docx]

**Selective blood-nerve-barrier leakiness with claudin-1 and vessel-associated-macrophages loss in diabetic polyneuropathy**

**Adel Ben-Kraiem^1#^, Reine-Solange Sauer^1#^, Carla Norwig^1^, Maria Popp^1^, Anna-Lena Bettenhausen^1^, Mariam Sobhy Atalla^1^, Alexander Brack^1^, Robert Blum^2^,^3^, Kathrin Doppler^3^, Heike L. Rittner^1^***

^1^ Center for Interdisciplinary Pain Medicine, Department of Anesthesiology, University Hospital of Würzburg, 97080 Würzburg, Germany

^2^ Institute of Clinical Neurobiology, University Hospital of Würzburg, 97078 Würzburg, Germany

^3^ Deparment of Neurology, University Hospital of Würzburg, 97080 Würzburg, Germany

^#^ These authors contributed equally

*Corresponding author: Heike Rittner, ORCID ID: 0000-0003-4867-0188 Center for Interdisciplinary Pain Medicine, Department Anesthesiology, University of Würzburg, Oberdürrbacher Strasse 6, D-97080 Würzburg, Germany, Phone: +49-170 7870047, Email: [rittner_h@ukw.de](mailto:rittner_h@ukw.de)

#

Supplementary Figures

**Suppl. Fig. 1 Unaffected thermal sensitivity in STZ induced diabetes.** Thermal nociceptive thresholds assessed by Hargreaves test before and 1-8 week after STZ application. All data points represent mean ± SEM. *P < 0.05 (n = 6). Two-way repeated measurement ANOVA and Bonferroni post-hoc test for multiple comparisons

**
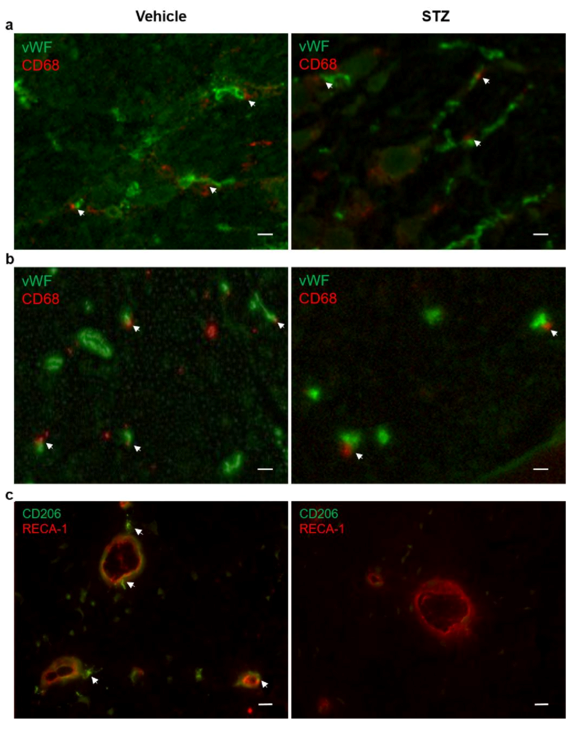
**

**Suppl. Fig. 2 Decreased number of vessel-associated-macrophages in sciatic nerve 8 weeks after STZ (full size of the inserts respectively Fig. 3i, and Fig. 7f and h)**. Staining for CD68 and von Willebrand factor in the DRG (a) and in the sciatic nerve (b). CD206 and RECA-1 immunostaining in sciatic nerve (c). Arrows depict representative examples, n = 6, a and b scale bar = 20 µm, c scale bar = 10 µm


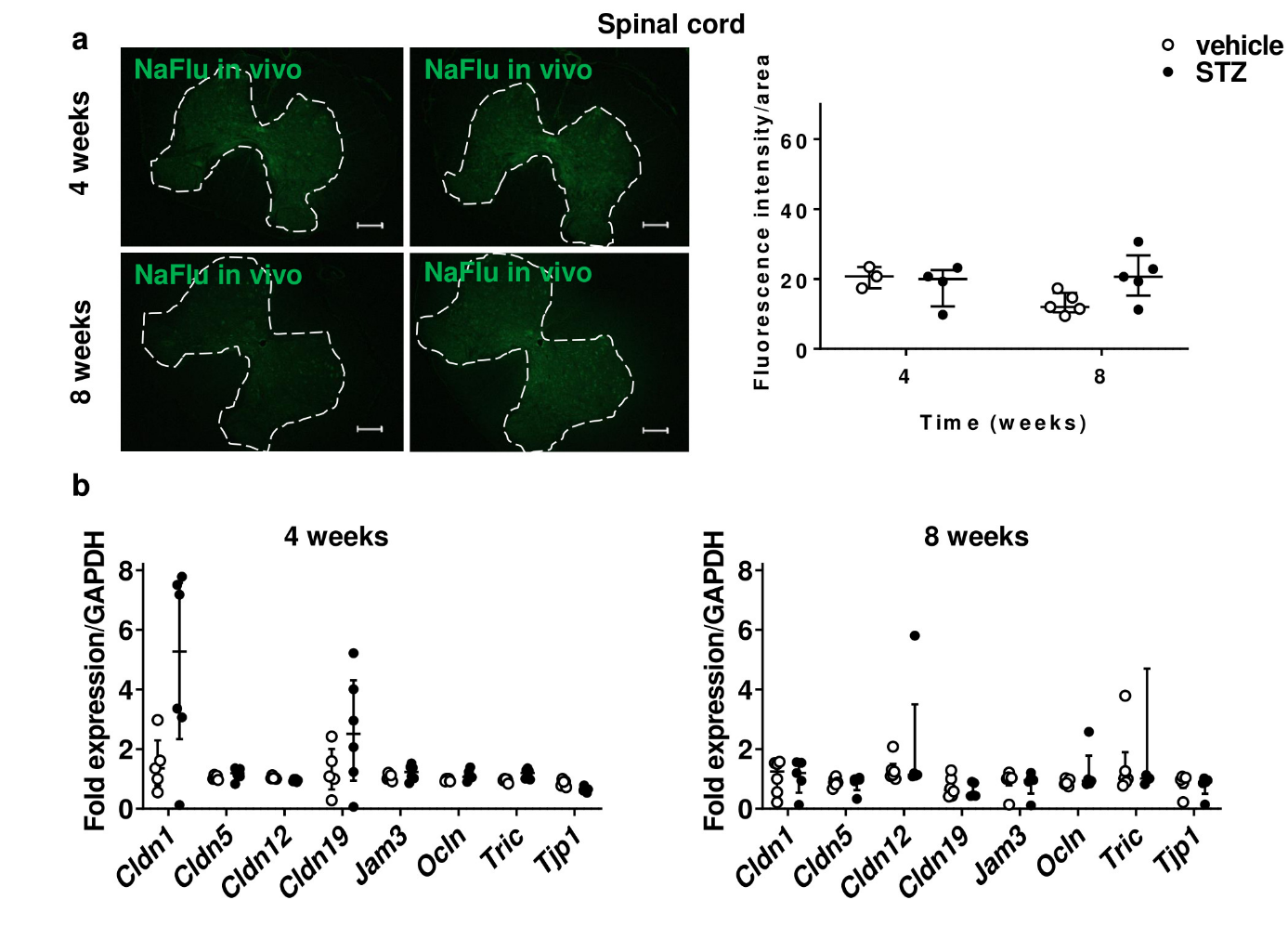


**Suppl. Fig. 3 Unchanged permeability to small molecules and unaffected tight junction proteins expression in the spinal cord in diabetic rats.** (**a**) Male Wistar rats were treated injected i.v. NaFlu injection 30 min before sacrifice. The spinal cord was harvested and sectioned for microscopy. Fluorescence and quantification of fluorescence intensity normalized by the stained area in spinal cord (n = 3-5). Scale bars = 100 µm, dot plots represent medians and interquartile range. (**b**) mRNA was extracted from entire spinal cord and tight junction protein mRNA quantified of *Cldn1*, *Cldn5*, *Cldn12*, *Cldn19*, *Jam3*, *Ocln*, *Tric* and *Tjp1* (ZO-1) (n = 6). Dot plots represent medians and interquartile range. A, B: Two-way ANOVA. P > 0.05

**
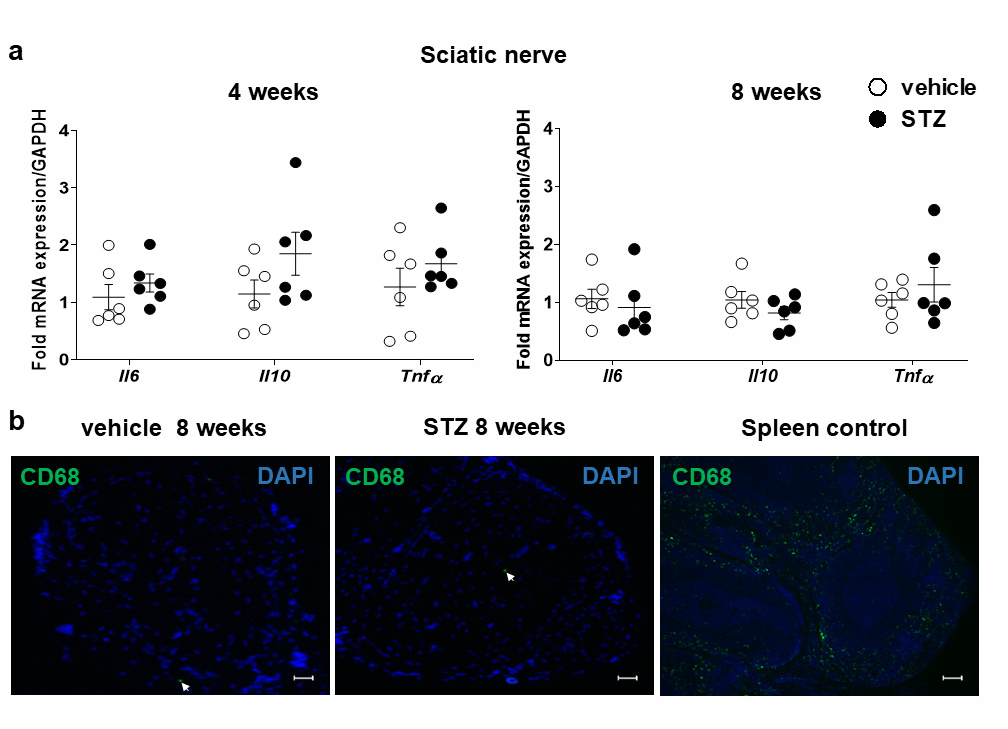
**

**Suppl. Fig. 4 Unaffected cytokine mRNA expression in entire sciatic nerve and no macrophages infiltration in sciatic nerve 8 weeks after STZ**. (**a**) Relative mRNA expression in sciatic nerve of *Il6*, *Il10* and *Tnfa* as analyzed with qPCR (n = 6). Dot plots and mean ± SEM. T-test was used for each comparison. (**b**) CD68 immunostaining in sciatic nerve and spleen. Arrows depict examples (representative example n = 3, scale bar = 100 µm)
